# DNA hypomethylation of Synapsin II CpG islands associates with increased gene expression in bipolar disorder and major depression

**DOI:** 10.1186/s12888-016-0989-0

**Published:** 2016-08-11

**Authors:** Cristiana Cruceanu, Elena Kutsarova, Elizabeth S. Chen, David R. Checknita, Corina Nagy, Juan Pablo Lopez, Martin Alda, Guy A. Rouleau, Gustavo Turecki

**Affiliations:** 1 McGill Group for Suicide Studies & Douglas Research Institute, McGill University, Montreal, QC Canada; 2 Montreal Neurological Institute, McGill University, Montreal, QC Canada; 3 Department of Psychiatry, Dalhousie University, Halifax, NS Canada; 4 Douglas Mental Health Institute, McGill University, 6875 LaSalle Blvd, Montreal, QC H4H 1R3 Canada

**Keywords:** Bipolar disorder, Major depressive disorder, DNA Methylation, Epigenetics, Mood disorders

## Abstract

**Background:**

The Synapsins (*SYN1*, *SYN2*, and *SYN3*) are important players in the adult brain, given their involvement in synaptic transmission and plasticity, as well as in the developing brain through roles in axon outgrowth and synaptogenesis. We and others previously reported gene expression dysregulation, both as increases and decreases, of Synapsins in mood disorders, but little is known about the regulatory mechanisms leading to these differences. Thus, we proposed to study DNA methylation at theses genes’ promoter regions, under the assumption that altered epigenetic marks at key regulatory sites would be the cause of gene expression changes and thus part of the mood disorder etiology.

**Methods:**

We performed CpG methylation mapping focusing on the three genes’ predicted CpG islands using the Sequenom EpiTYPER platform. DNA extracted from post-mortem brain tissue (BA10) from individuals who had lived with bipolar disorder (BD), major depressive disorder (MDD), as well as psychiatrically healthy individuals was used. Differences in methylation across all CpGs within a CpG island and between the three diagnostic groups were assessed by 2-way mixed model analyses of variance.

**Results:**

We found no significant results for *SYN1* or *SYN3*, but there was a significant group difference in *SYN2* methylation, as well as an overall pattern of hypomethylation across the CpG island. Furthermore, we found a significant inverse correlation of DNA methylation with *SYN2a* mRNA expression.

**Conclusions:**

These findings contribute to previous work showing dysregulation of Synapsins, particularly *SYN2*, in mood disorders and improve our understanding of the regulatory mechanisms that precipitate these changes likely leading to the BD or MDD phenotype.

**Electronic supplementary material:**

The online version of this article (doi:10.1186/s12888-016-0989-0) contains supplementary material, which is available to authorized users.

## Background

Synapsin genes – Synapsin I (*SYN1*), Synapsin II (*SYN2*), and Synapsin III (*SYN3*) – are interesting candidates for the etiology of mood disorders due to their involvement in the adult brain in synaptic transmission and plasticity, as well as in brain development in axon outgrowth and synaptogenesis [1]. Genetic association studies have linked SYN2 to epilepsy and schizophrenia, while brain expression studies have shown significant dysregulation in alcoholism, Huntington’s disease, schizophrenia and bipolar disorder [2]. A nonsense mutation in the *SYN1* gene was shown to cause familial epilepsy; and some studies have suggested an association of *SYN3* with schizophrenia [Reviewed in: [1, 2]].

Though the literature provides ample evidence for genetic associations and gene expression increases of Synapsins in mood disorders, little has been done to elucidate the regulatory mechanisms that lead to these disturbances. To date there is no evidence of altered DNA methylation of these genes in mood disorders; only one study of a single schizophrenia patient suggests potentially variably methylated sites in the distal *SYN3* promoter [3] and a recent study in rats proposes that early life stress, a precipitant of depressive behavior, leads to *SYN1* transcription start site hypermethylation and related mRNA downregulation in the amygdala [4].

Since we previously reported Synapsins mRNA up-regulation in mood disorders [5], we aimed to elucidate the regulatory mechanisms that may explain this by conducting a study of DNA methylation in putatively regulatory regions of the genes. DNA methylation at CpG islands has been most commonly associated to expression regulation – thus we chose to focus on *in silico* predicted islands at the proximal promoters of *SYN1* (845 bp) and *SYN2* (975 bp), as well as at a distal promoter of *SYN3* (613 bp) [2].

## Methods

Post-mortem prefrontal cortex (PFC) brain tissue from Brodmann Area 10 used in this study was obtained from the Douglas Bell-Canada Brain Bank (www.douglasbrainbank.ca) as described elsewhere [5, 6]. All procedures in this study were approved by the ethics review board of the Douglas Mental Health University Institute and informed consent for deposition in the brain bank and any resulting research was obtained from the next of kin. Clinical information, toxicology and history of psychoactive prescription drugs were collected for both cases and controls. Cases in this study were White Caucasian individuals with diagnoses of BD type I or type II (*n* = 13) or MDD (*n* = 18) and who died primarily by suicide. Controls were White Caucasian individuals with neither current nor past psychiatric diagnoses who died suddenly and could not have undergone resuscitation or other medical intervention (*n* = 14). We found no significant group differences in gender, age, brain pH or refrigeration delay (Additional file 1: Table S1).

**Fig. 1:**
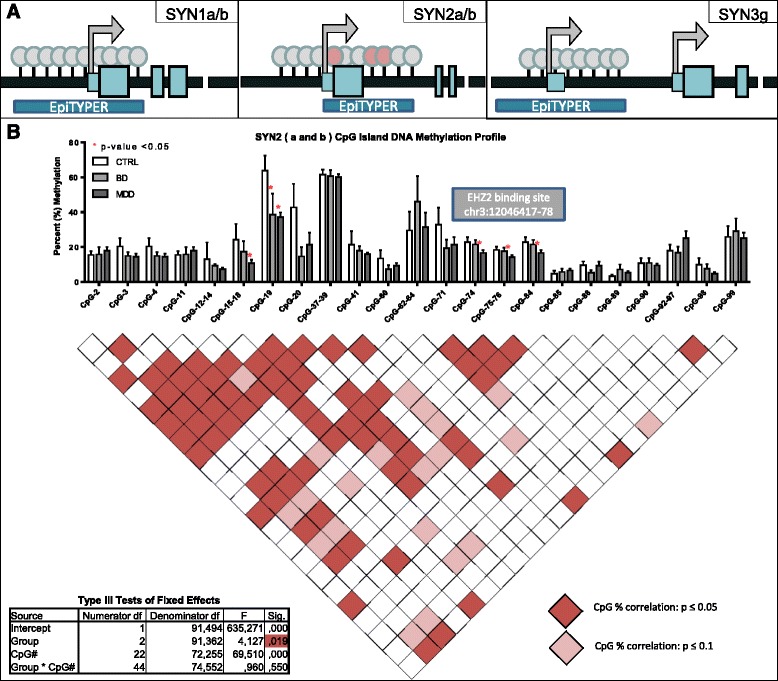
DNA methylation is decreased in individuals with mood disorders at the *SYN2* CpG island. **a** Graphical representations of the CpG islands predicted at the promoter and first exons of Synapsin genes. **b** Percent methylation levels for individual CpGs at *SYN2* show some significant (*p* < 0.05) or suggestive (*p* < 0.1) decreased levels of DNA methylation in BD and more in MDD, while several CpGs have no differences at all. The CpG-to-CpG correlation matrix shows some clusters of correlated percent methylation levels with others seemingly completely independent. The cluster at chr3:12046417-12046478, where 3 consecutive CpGs have decreased DNA methylation levels, overlaps with a predicted binding site for *EZH2*

We performed CpG methylation mapping focusing on the three Synapsins’ predicted CpG islands, which overlapped part of the promoter regions as well as the first exon and intron (Fig. 1a), using the EpiTYPER platform (Sequenom, San Diego, CA), as described previously [7]. Briefly, genomic DNA was extracted using the QIAamp DNA Mini Kit (QIAGEN, Toronto, CA) and concentrations and 260/280 ratios were determined using the Nanodrop 1000 spectrophotometer (Thermo Scientific). Genomic DNA from each sample was bisulfite-treated using the Epitect Bisulfite Kit (QIAGEN, Toronto, CA) following the manufacturer’s guidelines. Due to the mass threshold necessary for accurate measurement some CpGs (typically close together) were clustered and thus quantified together by the EpiTYPER software. Percent methylation levels were reported for each cluster or individual CpG.

Differences in methylation across all CpGs within a CpG island and between the three diagnostic groups were assessed by 2-way mixed model analyses of variance (ANOVA) with groups as the fixed factor and CpG dinucleotides percent methylation level as the repeated measure, followed by Fisher Least Significant Difference (LSD) post-hoc analyses. Since there was no difference between groups in regards to potential confounders: sex, brain pH, post-mortem delay, or age (Additional file 1: Table S1), no covariates were included in the mixed model. Because the EpiTYPER system is able to detect the methylation level in a mixture only as low as 5 %, we excluded all CpGs where methylation levels were below this threshold. Correlations between individual CpG percent methylation values were reported using Pearson’s correlation coefficients. In all cases, p-values were considered statistically significant at *p* ≤ 0.05 (labeled *) and suggestive at *p* ≤ 0.1 (labeled #).

## Results and discussion

In this study we explored the role of DNA methylation at promoter-region CpG islands mapping to the three synapsin genes: *SYN1*, *SYN2*, and *SYN3*. Given previous evidence of gene expression changes in the prefrontal cortex (PFC) we hypothesized that altered epigenetic marks at key regulatory sites would be in part responsible for this gene expression increase and thus part of the mood disorder etiology. We chose to work with the Brodmann Area 10 (BA10) given our previous findings regarding synapsin gene expression in this region, and more importantly its role in mood regulation shown through imaging studies as well as documented deficits in PFC-mediated working memory and executive function in BD [5, 8, 9]. The brain samples were obtained from the Douglas-Bell Canada Suicide brain bank, and as such the large majority of cases died by suicide while the controls died by sudden natural causes. A limitation of our study, and likely the larger part of postmortem mood disorder research, is that we cannot distinguish effects of the suicidal status from effects of the mood disorder itself. For this reason we made sure to include two diagnostic groups, and sought to identify differences between them that were unlikely to be a result of suicide.

We found no significant group differences for *SYN1* (*p* = 0.718) or *SYN3* (*p* = 0.449) (Additional file 2: Table S2), but there was a significant group effect for *SYN2* (*p* = 0.019, F = 4.127) (Additional file 2: Table S2; Fig. 1b inset). Given the positive result, we performed LSD post-hoc analyses for each CpG data point within the *SYN2* CpG island and found an overall pattern of hypomethylation across the island, with a few CpGs remaining completely unaltered (Fig. 1b). We found significant hypomethylation compared to controls in MDD at five loci and in BD at one locus (Fig. 1b, Additional file 3: Table S3). Due to the small differences between groups, no CpG passed corrections for multiple testing at a nominal p-value but all maintained suggestive significance (Bonferroni, *p*-value ≤ 0.1). The most interesting locus was at chr3:12046417-12046478, where three consecutive CpGs were significantly hypomethylated in MDD and their methylation levels strongly correlated across individuals (Fig. 1b, Pearson’s correlation matrix). In order to test whether there was a combined effect of the three CpG sites identified at this locus in MDD, we computed average CpG methylation values across the three sites and found a significant 25 % decrease in average methylation between MDD and control individuals at this locus (Additional file 4: Figure S1 Student’s T-Test, *p*-value = 0.03). This locus is part of a binding site for *EZH2* (Enhancer of Zeste homolog 2, or Enx1), a catalyst of mono- di- or tri-methylation of histone H3 at lysine 27 [10]. Thus the DNA hypomethylation we observed at this locus could interfere with recruitment of this factor and lead to altered H3K27 methylation levels. Unfortunately, our previous histone modification inquiries were limited to H3K4me3 [5] as is described in more detail later, but this finding suggests that it would be interesting to assess other histone modifications and their relationship to *SYN2* gene expression. Furthermore, EZH2 has been shown to also serve as a recruitment platform for DNA methyltransferases within the context of the Polycomb repressive complexes 2 and 3, and its decreased binding would lead to reduced DNA methylation [11]. This suggests that the hypomethyation observed could also be a result of EZH2 binding.

Previous work from our group showed significant mRNA up-regulation of the *SYN2a* isoform in BD and the *SYN2b* isoform in MDD [5]. Furthermore, the CpG island at *SYN2*, which is shared by both isoforms, was preceded by a region of significantly enriched H3K4me3 in MDD [5]. Since the same brain samples were used for the current study, we questioned whether our DNA methylation findings correlated with mRNA expression levels in these individuals. We computed the average methylation level for each individual and performed a Pearson’s correlation between these values and *SYN2a* and *SYN2b* mRNA expression as well as H3K4me3 enrichment and found a significant inverse correlation with *SYN2a* mRNA expression (*r* = -0.318, *p*-value = 0.014) and a suggestive inverse correlation with *SYN2b* mRNA expression (*r* = -0.264, *p*-value = 0.069). This is the expected direction, as promoter and first exon hypomethylation have been associated with increased gene expression [12]. There was no significant correlation with H3K4me3 enrichment, which is unexpected since a strong negative correlation was reported between DNA methylation and H3K4me3 at promoters and CpG islands [13]. On the other hand it is not necessarily surprising because the significant regions were not overlapping and it has been suggested that the correlation between DNA methylation and histone modifications may be genomic-region specific [14].

To our knowledge, this is the first study to show evidence of altered DNA methylation at any of the Synapsin genes in mood disorders; only one recent study in rats proposed that early life stress, a precipitant of depressive behavior, leads to *SYN1* transcription start site hypermethylation and related mRNA downregulation in the amygdala [4]. It would be of great interest to see other groups with access to similar post-mortem brain cohorts explore DNA methylation at the Synapsin loci. Furthermore, it would be interesting to see this work replicated in an analysis of single-cell or single-cell-type methylation since likely the fairly small effect sizes we identified here were in part due to cell-type heterogeneity within the tissue used for DNA extraction. This is a typical limitation of post-mortem brain epigenetic analysis, but incremental steps are currently being taken toward developing reliable methods for single-cell exploration [15–17].

Overall our current findings contribute to previous work showing dysregulation of *SYN2* in mood and other psychiatric disorders [18] and improve our understanding of the regulatory mechanisms that may explain differential expression of this gene. Although these results are of interest, regulatory mechanisms implicated in mood disorders are complex and dysregulation at multiple levels likely contributes to these complex phenotypes.

## Conclusions

In this work we showed evidence of decreased promoter DNA methylation which correlated with increased expression of Synapsin genes in mood disorders. This finding is important as it contributes to our understanding of the intricacies of mood disorder dysregulation at the genetic and epigenetic level in the brain.

## Additional files

Additional file 1: Table S1. Diagnostic group statistics and comparisons. No difference between potential confounders. Normality of each variable was computed per each diagnostic group. When the data were normally distributed, unpaired Student’s T-tests were performed, and when at least one group was not normally distributed (Shapiro-Wilk *p*-value ≤0.05), non-parametric tests were performed. Differences in Gender were tested with Fisher’s Exact tests. (XLSX 12 kb) Additional file 2: Table S2. Mixed Model ANOVA results for SYN1, SYN2, and SYN3 promoter CpG methylation. A. SYN1: Type III Tests of Fixed Effects. B. SYN2: Type III Tests of Fixed Effects. C. SYN3: Type III Tests of Fixed Effects. (XLSX 11 kb) Additional file 3: Table S3. Pairwise Comparisons for SYN2 CpG Methylation. (XLSX 15 kb) Additional file 4: Figure S1. DNA methylation (percent values) at significant individual CpGs. A-E. Scatter plots represent individual % methylation values for each of the 5 loci found to be significant for at least one diagnostic group (marked with * in Fig. 1) by Least Significant Difference (LSD) post-hoc analyses. F. Average % methylation for the three consequent CpGs shown to be significantly different in MDD compared to CTRL by Least Significant Difference (LSD) post-hoc analyses. The average values were found to be significantly different between the groups (Student’s T-Test, *p*-value = 0.03). (PPTX 354 kb)

## References

[1] Cesca F, Baldelli P, Valtorta F, Benfenati F (2010). The synapsins: key actors of synapse function and plasticity. Prog Neurobiol.

[2] Cruceanu C, Freemantle E, Alda M, Rouleau GA, Turecki G (2013). Epigenetic regulation of synapsin genes in mood disorders. Neuropsychopharmacology.

[3] Murphy BC, O’Reilly RL, Singh SM (2008). DNA methylation and mRNA expression of SYN III, a candidate gene for schizophrenia. BMC Med Genet.

[4] Park HJ, Kim SK, Kang WS, Chung JH, Kim JW (2014). Increased activation of synapsin 1 and mitogen-activated protein kinases/extracellular signal-regulated kinase in the amygdala of maternal separation rats. CNS Neurosci Ther.

[5] Cruceanu C, Alda M, Nagy C, Freemantle E, Rouleau GA, Turecki G (2013). H3K4 tri-methylation in synapsin genes leads to different expression patterns in bipolar disorder and major depression. Int J Neuropsychopharmacol.

[6] Lopez de Lara C, Jaitovich-Groisman I, Cruceanu C, Mamdani F, Lebel V, Yerko V, Beck A, Young LT, Rouleau G, Grof P (2010). Implication of synapse-related genes in bipolar disorder by linkage and gene expression analyses. Int J Neuropsychopharmacol.

[7] Gross JA, Fiori LM, Labonte B, Lopez JP, Turecki G (2013). Effects of promoter methylation on increased expression of polyamine biosynthetic genes in suicide. J Psychiatr Res.

[8] Robinson JL, Monkul ES, Tordesillas-Gutierrez D, Franklin C, Bearden CE, Fox PT, Glahn DC (2008). Fronto-limbic circuitry in euthymic bipolar disorder: evidence for prefrontal hyperactivation. Psychiatry Res.

[9] Malhi GS, Ivanovski B, Hadzi-Pavlovic D, Mitchell PB, Vieta E, Sachdev P (2007). Neuropsychological deficits and functional impairment in bipolar depression, hypomania and euthymia. Bipolar Disord.

[10] Schwartz YB, Pirrotta V (2007). Polycomb silencing mechanisms and the management of genomic programmes. Nat Rev Genet.

[11] Vire E, Brenner C, Deplus R, Blanchon L, Fraga M, Didelot C, Morey L, Van Eynde A, Bernard D, Vanderwinden JM (2006). The Polycomb group protein EZH2 directly controls DNA methylation. Nature.

[12] Brenet F, Moh M, Funk P, Feierstein E, Viale AJ, Socci ND, Scandura JM (2011). DNA methylation of the first exon is tightly linked to transcriptional silencing. PLoS One.

[13] Liu H, Chen Y, Lv J, Liu H, Zhu R, Su J, Liu X, Zhang Y, Wu Q (2013). Quantitative epigenetic co-variation in CpG islands and co-regulation of developmental genes. Sci Rep.

[14] Yan H, Zhang D, Liu H, Wei Y, Lv J, Wang F, Zhang C, Wu Q, Su J, Zhang Y (2015). Chromatin modifications and genomic contexts linked to dynamic DNA methylation patterns across human cell types. Sci Rep.

[15] Schwartzman O, Tanay A (2015). Single-cell epigenomics: techniques and emerging applications. Nat Rev Genet.

[16] Angermueller C, Clark SJ, Lee HJ, Macaulay IC, Teng MJ, Hu TX, Krueger F, Smallwood SA, Ponting CP, Voet T (2016). Parallel single-cell sequencing links transcriptional and epigenetic heterogeneity. Nat Methods.

[17] Hyun BR, McElwee JL, Soloway PD (2015). Single molecule and single cell epigenomics. Methods.

[18] Molinaro L, Hui P, Tan M, Mishra RK (2015). Role of presynaptic phosphoprotein synapsin II in schizophrenia. World J Psychiatry.

