# Supplementary figures and images for: DNA hypomethylation of Synapsin II CpG islands associates with increased gene expression in bipolar disorder and major depression
Source: BMC Psychiatry. 2016 Aug 11;16:286. doi: 10.1186/s12888-016-0989-0 (PMC4982122; doi:10.1186/s12888-016-0989-0)

## Slide 1
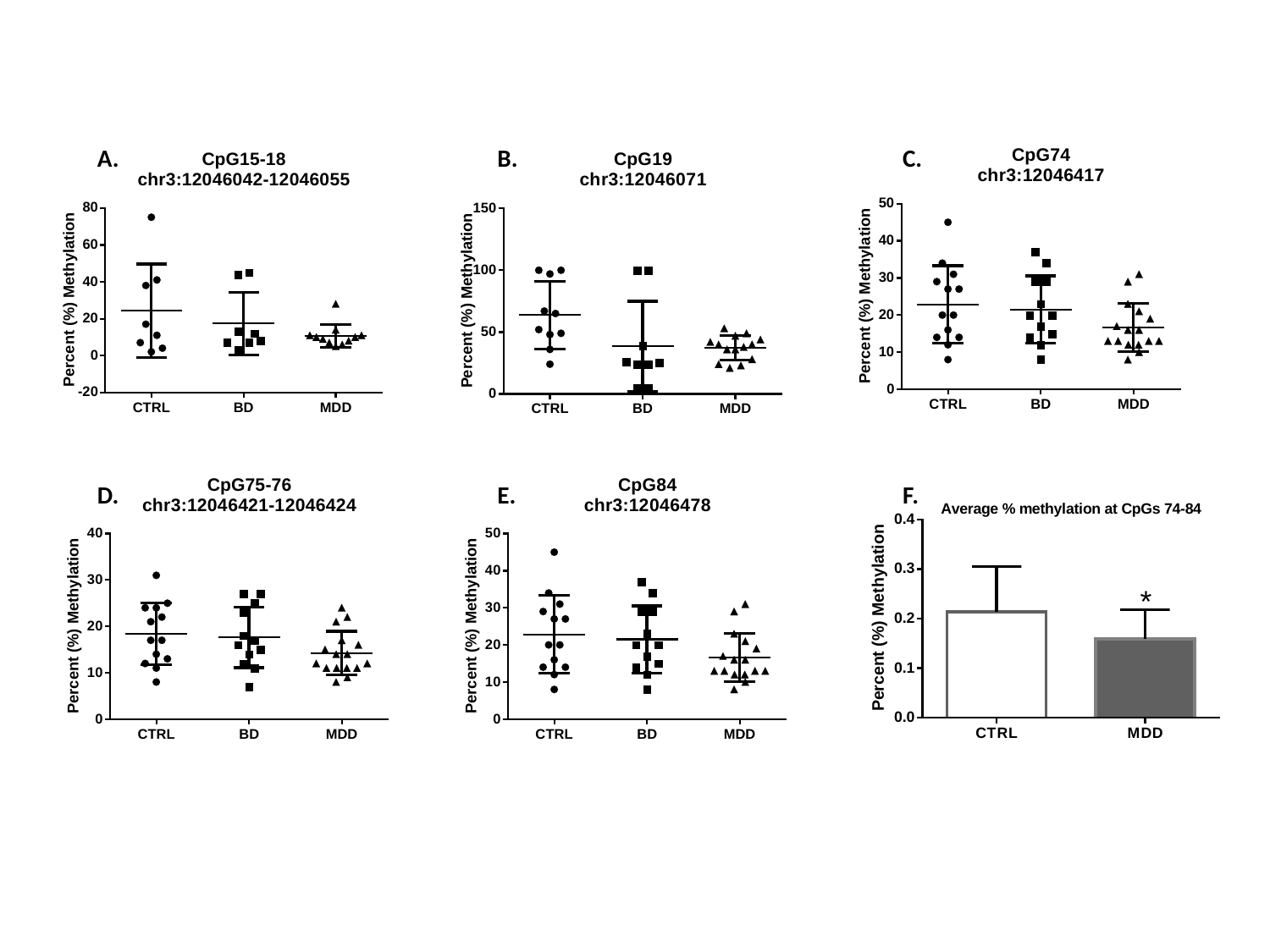

C.
A.
B.
D.
E.
F.

Supplement: Additional file 4: Figure S1. — DNA methylation (percent values) at significant individual CpGs. A-E. Scatter plots represent individual % methylation values for each of the 5 loci found to be significant for at least one diagnostic group (marked with * in Fig. 1) by Least Significant Difference (LSD) post-hoc analyses. F. Average % methylation for the three consequent CpGs shown to be significantly different in MDD compared to CTRL by Least Significant Difference (LSD) post-hoc analyses. The average values were found to be significantly different between the groups (Student’s T-Test, p-value = 0.03). (PPTX 354 kb) [file 12888_2016_989_MOESM4_ESM.pptx]
